# Clinical outcomes of proximal gastrectomy with gastric tubular reconstruction and total gastrectomy for proximal gastric cancer: A matched cohort study

**DOI:** 10.3389/fsurg.2022.1052643

**Published:** 2023-01-12

**Authors:** Jingxiao Fu, Yi Li, Xuechao Liu, Xuelong Jiao, Yuhao Wang, Hongyu Qu, Zhaojian Niu

**Affiliations:** Department of Gastrointestinal Surgery, Affiliated Hospital of Qingdao University, QingDao, China

**Keywords:** proximal gastrectomy, gastric tubular reconstruction, reflux esophagitis, nutritional status, hemoglobin

## Abstract

**Background:**

Proximal gastrectomy with gastric tubular reconstruction is a surgical procedure that can preserve function in patients with proximal gastric cancer. However, whether gastric tubular reconstruction with proximal gastrectomy has certain advantage in some aspects over total gastrectomy is controversial. To evaluate the benefit of gastric tubular reconstruction after proximal gastrectomy, we compared gastric tubular reconstruction with total gastrectomy for proximal gastric cancer.

**Method:**

A total of 351 patients were enrolled. Concurrent total gastrectomy patients matched with the Proximal gastrectomy group in age, sex, body mass index, clinical stage, and ASA score were selected by propensity score matching. Preoperative basic information, perioperative indicators, histopathological features, postoperative complications and nutritional status, reflux were compared between the two groups.

**Results:**

There was no significant difference in the incidence of reflux between two groups (14.8% and 6.5% respectively, *P* = 0.085). There were significant differences between the two groups in bowel function recovery (2.29 ± 1.16 vs. 3.01 ± 1.22; *P* = 0.039) and start of soft diet (4.06 ± 1.81 vs. 4.76 ± 1.69; *P* = 0.047). There were no significant differences between the two groups in nutritional status one year after surgery. However, the decrease in serum hemoglobin in the TG group at 3 and 6 months after surgery was significantly higher than that in the PG group (*P* = 0.032 and 0.046, respectively). One month after surgery, %BW loss in TG group was significantly lower than that in the PG group (*P* = 0.024).

**Conclusion:**

The Proximal gastrectomy group has better clinical outcome and gastric tubular reconstruction is simple, similar complications and reflux rates, gastric tubular reconstruction may be more suitable for proximal gastric cancer.

## Introduction

The incidence of proximal gastric cancer is increasing worldwide (1–3). Proximal gastric cancer can occur in the upper 1/3 of the stomach or in the middle part of stomach; it constitutes more than 30% of gastric cancer cases. According to the Japan Gastric Cancer Association, the incidence of proximal gastric cancer increased by 0.8% from 2002 to 2011 (4). According to the 5th edition of the Japanese Guidelines for the Treatment of Gastric Cancer, total gastrectomy is the standard surgical treatment for upper 1/3 gastric cancer or AEG (5). The advantage of this surgical approach lies in the thorough dissection of lymph nodes that may metastasize and the avoidance of esophagogastric reflux complications. However, after total gastrectomy, patients will inevitably have nutritional metabolic disorders, especially patients with early proximal gastric cancer, which are more prominent (6–8). After total gastrectomy, the storage, mechanical grinding, secretion and other functions of the stomach are permanently lost, resulting in postoperative malnutrition, including decreased postoperative body mass, anemia, diarrhea, and dumping syndrome (8, 9).

Proximal gastrectomy of antireflux anatomical structures at the esophagogastric junction, including the cardia and His Angle, with simultaneous separation of the vagus nerve, resulted in an increased incidence of pyloric spasm and obstruction of residual stomach emptying (10). Some patients developed reflux esophagitis after surgery, which seriously affected the quality of life. Studies have shown that the incidence of postoperative reflux esophagitis is approximately 50% when traditional esophagogastrostomy is used in proximal gastrectomy (11, 12). To prevent RE, several reconstructive procedures after PG have been reported, such as double-flap (13), double-tract (14, 15), and jejunal interposition (16). However, these techniques are complicated, time-consuming and sometimes unsatisfactory.

Esophagogastrostomy was described by Shiraishi et al. in 1998 for the treatment of early proximal gastric cancer (17). This method excises part of the gastric antrum, reduces gastrin and gastric acid secretion, and objectively reduces reflux substances. After anastomosis, the tube stomach can make food pass through quickly and avoid food retention. The top of the residual stomach is similar to the fundus of the stomach, which can buffer the upward reflux of gastric juice and temporarily store the reflux of gastric juice. Therefore, the operation method has a good antireflux effect. Some research results show that compared with traditional residual gastroesophageal anastomosis, esophageal gastric tube anastomosis has a better quality of life for patients (18).

The purpose of this study was to determine whether gastric tubular reconstruction is a viable option after PG in terms of postoperative reflux and some nutritional indicators. We conducted a retrospective matched cohort study comparing the effects of gastric tubular reconstruction and total gastrectomy on patients with proximal gastric cancer.

## Methods

All patients with upper one-third gastric cancer consecutively received surgical treatment in the Gastrointestinal Surgery Department of Qingdao University Affiliated Hospital from January 2017 to February 2021. Upper third gastric cancer is defined as adenocarcinoma of the upper third of the stomach, with or without esophagogastric junction adenocarcinoma, according to the Classification of the Japan Gastric Cancer Association (JGCA) (19). The location of the primary carcinoma was determined by esophagogastroscopy. Inclusion criteria were that all patients had tumors located in the upper third of the stomach, clinical stage (CT1N0-1M0/CT2-3N0M0), R0 resection, and age 20–80. Exclusion criteria were neoadjuvant therapy, any malformation or ulcerative scarring of the distal stomach or duodenum, severe heart, lung, liver, kidney disease or mental abnormalities, and double primary carcinoma.

Patients were divided into two groups based on whether they underwent total or proximal gastrectomy. All patients underwent R0 resection. Patients underwent propensity score matching analysis, which adjusted for five factors, namely, age, sex, body mass index (BMI), pathological stage, and American Society of Anesthesiologists (ASA) body condition score, to offset selection bias. The matched whole stomach group was compared with the proximal stomach group based on demographic, clinical, surgical, and pathological features, postoperative outcomes (including early and late complications), postoperative nutritional status, and reflux esophagitis and reflux symptoms at endoscopic examination 1 year after surgery.

Preoperative tumor staging was assessed by computed tomography and gastroscopy. T stage and N stage were determined using the latest AJCC/UICC TNM staging system (20), and histological types were consistent with the Japanese classification of gastric cancer (19).

### Surgical procedure for PG with gastric tubular reconstruction and TG

**Figure 1:**
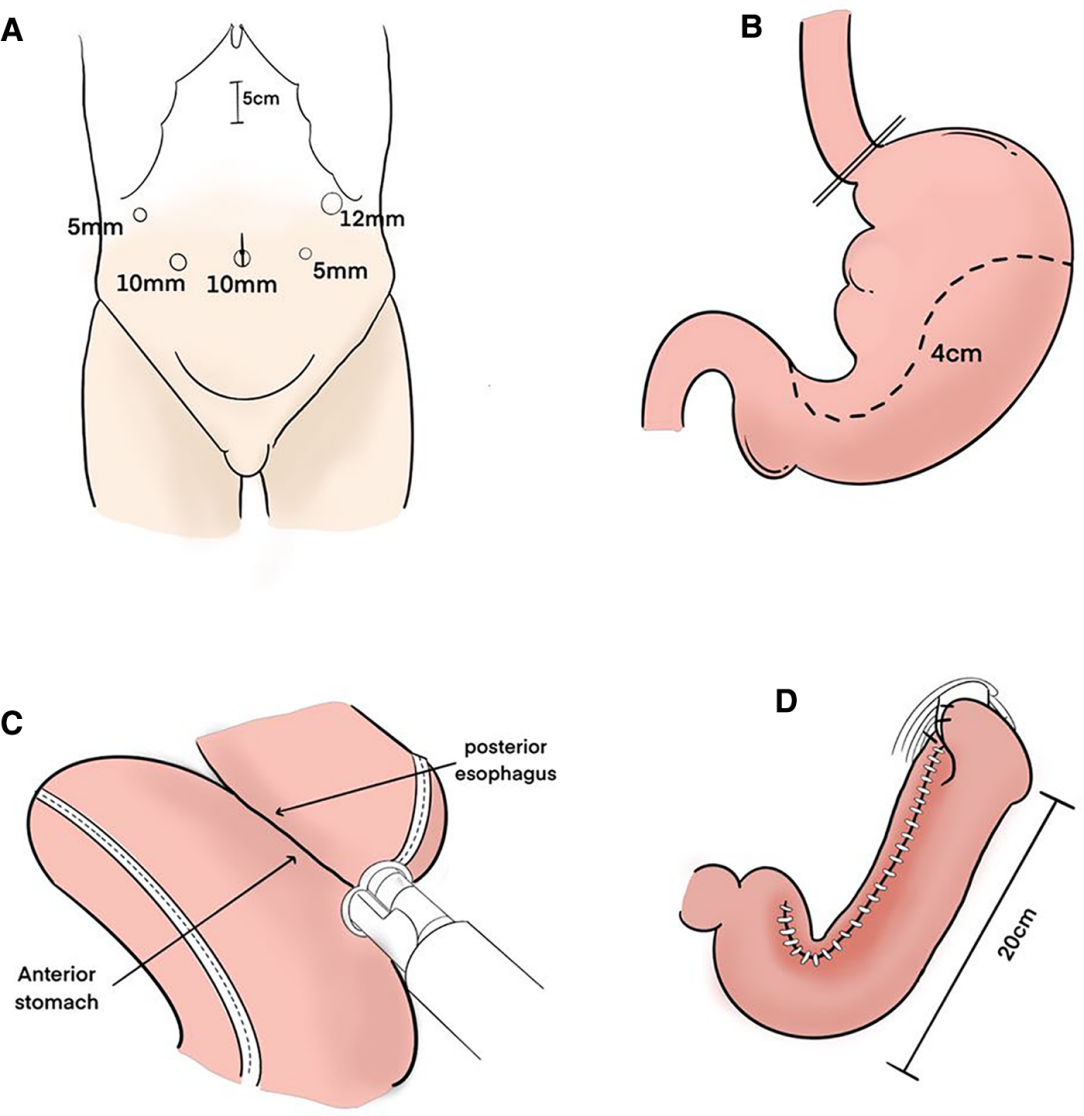
Surgical procedure. (**A**) Trocar sites. (**B**) After the esophagus was transected, the upper part of the stomach was excised along a dashed line to create a gastric tube. Its width is about 4 cm. (**C**) The anterior wall of the gastric tube and the posterior wall of the esophagus were anastomosed with linear stapler. (**D**) After anastomosis was performed, the entry hole was closed with barbed suture. The gastric tube was anchored with the right and left crus of the diaphragm by one stitch each to prevent hiatus hernia. The length of the whole gastric tube on the greater curvature side is about 20 cm.

**Figure 2:**
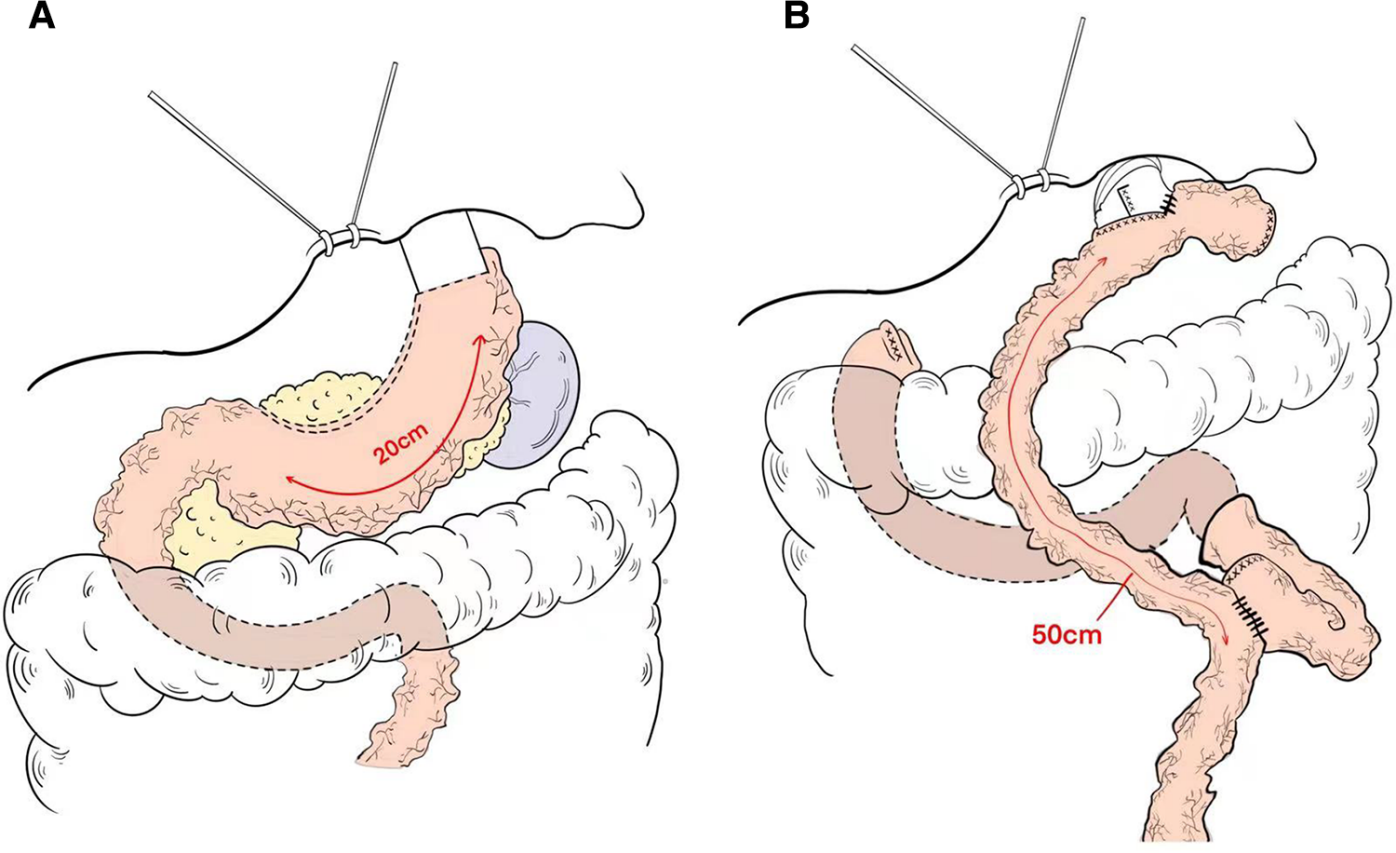
Schema of reconstruction after (**A**) proximal gastrectomy with tube gastric and (**B**) total gastrectomy with roux-en-Y esophagojejunostomy reconstruction.

All surgeries were performed by three upper gastrointestinal specialists using the same procedure (Figures 1, 2).

Five ports were introduced, as shown in Figure 1A. Before reconstruction, lymph node dissection was completed according to the Japanese guidelines (5). Open the diaphragm angle, and bare the lower end of the esophagus by 2–3 cm. The esophagus was transected about the cardia with an endo GIA stapler. A 5 cm incision was made in the middle of the upper abdomen, and the stomach was extruded through it. The stomach was marked with a dotted line using a sterile marker and ruler. Approximately 5 cm below the tumor, the upper part of the stomach was excised along a dashed line to make a gastric tube (Figure 1B). The pneumoperitoneum was re-established. An entry hole was made on the posterior side of the esophagus stump and on the anterior wall of the gastric tube 40 mm distal from the proximal stump laparoscopically. Then, the linear stapler was applied between the anterior wall of the gastric tube and the posterior wall of the esophagus laparoscopically (Figure 1C). After anastomosis was performed, the entrance hole was enclosed with stitches. The gastric tube was anchored with the right and left crus of the diaphragm by one stitch each to prevent hiatus hernia (Figure 1D). The vagus nerve was not preserved.

The length of the gastric tube is generally approximately 20 cm and the width is approximately 4 cm, which indicates: (1) The size of the residual stomach was correlated with gastric emptying; the larger the stomach, the worse the emptying; (2) Residual gastric emptying is related to gravity, intragastric pressure and residual gastric compliance; (3) The smaller the gastric tube, the worse its compliance is and the less likely it is to have reflux. The gastric tube after anastomosis with the esophagus is shown in (Figure 2A).

Reconstruction technique of esophagojejunostomy, the jejunum was transected at a distance of 20 cm from the Treus ligament, and a tube stapler was placed at a distance of 50 cm from the distal end by purse-string suture. The jejunum was anastomosed laterally through the proximal jejunum, and the stump was closed by GIA stapler, and the absorbable suture was embedded. The pneumoperitoneum was re-established, and the esophagus and the distal jejunum were punctured. The lateral jejunum anastomosis of the posterior esophageal wall was completed by GIA stapler, and the common opening was closed by continuous suture of the barb line. Esophagojejunostomy (Roux-en-Y) was performed after total gastrectomy (Figure 2B).

### Data collection and evaluation of outcomes

Patients are enrolled in this study, three data management staff members will be assigned to collect relevant data. The basic characteristics of patients collected before surgery were age, sex, body mass index, ASA score, hematologic indices, serum tumor markers, FEV1 and Her2 expression. The clinicopathological features were TNM stage, tumor size and location, Lauren classification, and tumor cell differentiation. The operation was characterized by operative time, estimated blood loss, pathological proximal and distal margins, and number of lymph nodes removed. Postoperative outcomes were mean maximum body temperature during the first 3 days, analgesic use 1–5 days after surgery, days of bowel function recovery, time to start soft diet, postoperative hospital stay, and early complications (within 30 days after surgery). Patients were evaluated 1, 3, 6 and 12 months after operation, and their characteristics and results were obtained by viewing electronic medical records and picture archiving and communication systems. Postoperative morbidity was described based on the Clavien-Dindo classification of JOCG criteria for postoperative complications and according to the General Terminology criteria for Adverse Events (CTCAE 5.0) (21–23). Clinical features and nutritional status 1 year after operation were evaluated by PG-SGA (24).

Follow-up results 1 year postoperatively were based on the Visick classification (Grade i: asymptomatic; Grade ii: mild symptoms without medication; Grade iii: mild symptoms that are easily controlled by medication; Grade iiii: severe symptoms, the duration or surgery) assessment of reflux symptoms and endoscopic findings were scored in terms of the Los Angeles Classification of reflux esophagitis (25). The Visick score and endoscopy results were obtained in the clinic. All endoscopy results were graded by the same surgeon according to the Los Angeles Classification System.

Nutritional parameters after gastrectomy were assessed on the basis of changes in serum prealbumin, albumin, hemoglobin, prognostic nutritional index (PNI) and the percentage of BW loss (%BW loss) at 1, 3, 6, and 12 months after surgery (26). The percentage of BW loss (%BW loss) was calculated as follows: %BW loss = (BW at 1/3/6/12 months after surgery−preoperative BW)/(preoperative BW × 100). PNI was calculated using the following formula: 10 × serum albumin value (g/dl) + 0.005 × lymphocyte count in peripheral blood (27). On the CT images, the cross-sectional area of the psoas muscle was measured at the level of the third lumbar vertebra (L3). Psoas muscle index (PMI) = (Area of the psoas muscle at L3 [cm]2)/(height[m]2). %PMI loss was all defined in the same way as %BW loss (28, 29).

### Statistical analysis

Propensity score matching was based on gender, age, body mass index, clinical stage, and ASA score. Continuous variables of normal distribution were expressed by (*X* ± *S*), and comparison between two groups was compared by *T* test. Non-normally distributed continuous variables were represented by median (range), and comparisons between the two pairs were performed using Wilcoxon signed rank test. The categorical variables were expressed in terms of number of cases and percentage, and comparisons between the two groups were performed by Chi-square test or Fisher precise test. All analyses were performed using SPSS 26.0. (*P* < 0.05) was considered statistically significant.

### Ethics statement

The data for this study were collected in the course of general clinical practice, so informed consent signed by each patient was obtained for any surgical and clinical procedure. This protocol is in line with the ethical guidelines of the World Medical Association Declaration of Helsinki adopted by the 18th World Medical Association Congress held in Helsinki, Finland in June 1964. Institutional Review board approval is not required. Since this study was retrospective, patients’ consent was not required for inclusion in the study.

## Results

### Patient characteristics

**Figure 3:**
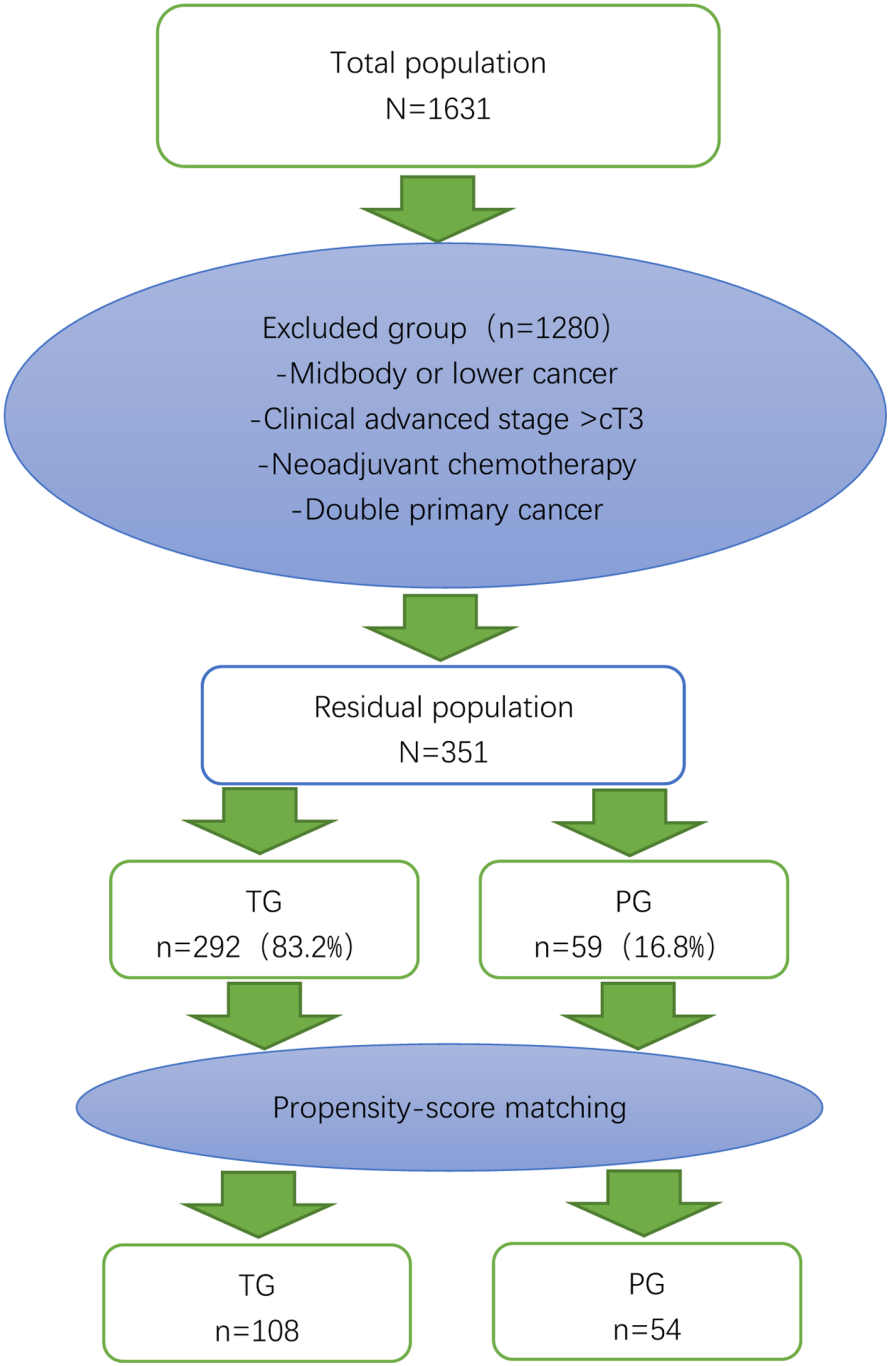
Flow chart of patient selection and propensity score matching. The 54 patients who underwent proximal gastrectomy (PG) were matched to 108 patients who underwent total gastrectomy (TG) in terms of age, sex, body mass index, clinical stage, and American Society of Anesthesiologists (ASA) score.

A total of 1,631 gastric cancer patients underwent surgery (Figure 1). Of those, 1,178 were excluded because the tumor was in the middle or lower part of the stomach. Of the remaining 453 patients, patients with advanced cancer (*n* = 51), neoadjuvant chemotherapy (*n* = 48) and dual primary cancer (*n* = 3). 59 patients received proximal gastrectomy and 292 patients received total gastrectomy. After 1:2 matching between the PG group and TG group, there were 54 patients in the PG group and 108 patients in the TG group (Figure 3 and Table 1).

**Table 1:** Patient characteristics.

Factor	Entire cohort		*P*	Matched cohort		*P*
	PG (*n* = 59)	TG (*n* = 292)		PG (*n* = 54)	TG (*n* = 108)	
Age, year, median (range)	63 (34–79)	64 (29–78)	0.524	65 (41–74)	64 (39–73)	0.879
Sex			0.096			0.303
Male, *n* (%)	42 (71.2)	236 (80.8)		38 (70.4)	84 (77.8)	
Female, *n* (%)	17 (28.8)	56 (19.2)		16 (29.6)	24 (22.2)	
BMI, Kg/m^2^ ± SD	25.17 ± 3.24	24.35 ± 3.73	0.117	25.27 ± 3.08	24.91 ± 3.38	0.512
ASA physical status			0.322			0.480
0–1, *n* (%)	21 (35.6)	85 (29.1)		20 (37.0)	34 (31.5)	
≥2, *n* (%)	38 (64.4)	207 (70.9)		34 (63.0)	74 (68.5)	
Preoperative Hb, g/L ± SD	134.09 ± 14.93	131.01 ± 15.81	0.169	135.17 ± 15.14	132.46 ± 15.36	0.289
Preoperative albumin, g/L ± SD	43.01 ± 2.11	42.23 ± 2.43	0.022	42.57 ± 2.59	41.82 ± 2.85	0.106
Psoas muscle index, cm^2^/m^2^ ± SD	169.48 ± 33.09	168.49 ± 35.21	0.842	168.78 ± 32.13	167.52 ± 33.17	0.818
Lymphocyte count, 10^9^/L ± SD	1.43 ± 0.39	1.46 ± 0.41	0.606	1.41 ± 0.45	1.44 ± 0.49	0.706
Preoperative pre-albumin, g/L ± SD	242.49 ± 36.02	234.41 ± 40.63	0.157	244.37 ± 35.13	237.09 ± 39.02	0.249
Preoperative creatinine, umol/L ± SD	61.03 ± 14.91	63.01 ± 15.17	0.360	62.98 ± 15.09	63.57 ± 13.31	0.800
Previous treatment with ESD, *n* (%)	8 (13.6)	14 (4.8)	0.011	8 (14.8)	14 (12.9)	0.746
NRS 2002 Score			0.406			0.652
<3, *n* (%)	36 (61.0)	161 (55.1)		33 (61.1)	62 (57.4)	
≥3, *n* (%)	23 (39.0)	131 (44.9)		21 (38.9)	46 (42.6)	
CCI score			0.712			0.737
0–2, *n* (%)	27 (45.8)	126 (43.2)		25 (46.3)	47 (43.5)	
≥3, *n* (%)	32 (54.2)	166 (56.8)		29 (53.7)	61 (56.5)	
Her2			0.391			0.554
0, *n* (%)	43 (72.9)	208 (71.2)		40 (74.1)	78 (72.2)	
+, *n* (%)	10 (16.9)	58 (19.9)		9 (16.7)	20 (18.5)	
++, *n* (%)	1 (1.7)	14 (4.8)		1 (1.8)	6 (5.6)	
+++, *n* (%)	5 (8.5)	12 (4.1)		4 (7.4)	4 (3.7)	
AFP, ng/ml median (IQR)	3.02 (2.28)	2.88 (1.94)	0.449	2.78 (2.21)	2.91 (1.95)	0.473
CEA, ng/ml median (IQR)	2.51 (2.74)	2.76 (3.08)	0.323	2.47 (2.63)	2.67 (2.99)	0.178
CA-199, U/ml median (IQR)	9.51 (9.68)	10.56 (15.11)	0.114	9.58 (9.81)	11.12 (15.02)	0.137
CA-125, U/ml median (IQR)	10.32 (6.40)	9.78 (6.33)	0.108	9.81 (6.63)	9.94 (7.12)	0.998
CA-724, U/ml median (IQR)	1.65 (2.36)	2.32 (3.70)	0.142	1.67 (2.42)	2.09 (3.10)	0.221
CA-242, U/ml median (IQR)	5.07 (4.09)	5.44 (7.89)	0.062	5.13 (4.70)	5.59 (8.39)	0.098
History of smoking, *n* (%)	31 (55.9)	174 (59.6)	0.317	29 (53.7)	66 (61.1)	0.367
FEV1.0, % ± SD	78.1 ± 10.3	77.3 ± 9.8	0.417	78.3 ± 10.6	77.6 ± 12	0.536
Number of comorbidities			0.640			0.884
0, *n* (%)	24 (40.7)	101 (34.6)		22 (40.7)	39 (36.1)	
1, *n* (%)	27 (45.8)	157 (53.8)		25 (46.3)	56 (51.9)	
2, *n* (%)	6 (10.2)	23 (7.9)		5 (9.3)	10 (9.3)	
3, *n* (%)	2 (3.4)	11 (3.8)		2 (3.7)	3 (2.8)	
Comorbidities			0.996			0.935
Hypertension	21 (35.6)	109 (37.3)		19 (35.2)	42 (38.9)	
Diabetes	12 (20.3)	66 (22.6)		10 (18.5)	24 (22.2)	
Hepatic disease	1 (1.7)	5 (1.7)		1 (1.9)	2 (1.9)	
Cardiac disease	4 (6.8)	19 (6.5)		2 (7.4)	5 (4.6)	
Cerebrovascular disease	4 (6.8)	16 (5.5)		1 (5.6)	4 (3.7)	
Asthma	1 (1.7)	5 (1.7)		1 (1.9)	2 (1.9)	
History of pulmonary tuberculosis	1 (1.7)	4 (1.4)		0	1 (0.9)	

The basic clinical characteristics of patients in the two groups were shown in Table 1. In the whole cohort, the number of patients who underwent ESD before surgery in the PG group was significantly greater than that in the TG group (*P* = 0.011), and there was no significant difference in preoperative hematological nutritional indicators, tumor markers or complications. The matched baseline features are well balanced.

### Perioperative clinical outcomes

In the entire cohort, the two groups were on a liquid diet (4.11 ± 1.77 in PG vs. 4.91 ± 1.89; *P* = 0.032) and showed bowel function recovery (2.77 ± 1.25 in PG vs. 3.44 ± 1.62 in TG; *P* = 0.024). The blood loss and operation time in the PG group were lower than those in TG group, but there were no significant differences (*P* > 0.05). There was no statistically significant difference in medical cost or 30-day readmission between the two groups (*P* > 0.05). After propensity score matching, the start of a soft diet after the operation in the PG group was 0.7 days sooner than in the TG group (4.06 ± 1.81 vs. 4.76 ± 1.69; *P* = 0.047), and the recovery time of bowel function in the PG group was 0.72 days shorter than in the TG group (2.29 ± 1.16 vs. 3.01 ± 1.22; *P* = 0.039). In the matched cohort, there were no statistically significant differences in the length of postoperative hospital stay, the average maximum body temperature in the first three days after surgery, and the number of patients using analgesics 1–5 days after surgery (*P* > 0.05) (Table 2).

**Table 2:** Perioperative indicators.

Factor	Entire cohort		*P*	Matched cohort		*P*
	PG (*n* = 59)	TG (*n* = 292)		PG (*n* = 54)	TG (*n* = 108)	
Operation time (min ± SD)	166.41 ± 41.78	179.51 ± 42.84	0.032	165.32 ± 42.23	177.61 ± 42.96	0.086
Estimated blood loss (ml ± SD)	57.19 ± 27.81	64.86 ± 30.03	0.071	55.66 ± 4.49	57.21 ± 5.13	0.061
Operation method			0.120			0.081
Laparoscopic, *n* (%)	44 (74.6)	187 (64.0)		40 (74.1)	65 (60.2)	
Robotic, *n* (%)	15 (25.4)	105 (36.0)		14 (25.9)	43 (39.8)	
Lymph node dissection, *n* (%)			<0.001			<0.001
D1+	58 (98.3)	81 (27.7)		54 (100)	62 (57.4)	
D2	1 (1.7)	211 (72.3)		0	46 (42.6)	
Combined resection			0.786			>0.999
Gallbladder	2	6		1	2	
Spleen	0	3		0	1	
Bowel function recovery (days ± SD)	2.77 ± 1.25	3.19 ± 1.31	0.024	2.29 ± 1.16	2.71 ± 1.22	0.037
Start of soft diet (days ± SD)	4.11 ± 1.77	4.68 ± 1.89	0.033	4.06 ± 1.81	4.66 ± 1.69	0.039
Analgesic use on Post-operative day 1-5, *n* (%)	32 (54.2)	167 (57.2)	0.773	29 (53.7)	61 (56.5)	0.867
Body temperature during the first 3 days^a^						
Post-operative day 1 (mean ± SD)	37.6°C ± 1.7°C	37.8°C ± 1.9°C	0.454	37.7°C ± 1.9°C	37.9°C ± 1.7°C	0.498
Post-operative day 2 (mean ± SD)	37.2°C ± 1.4°C	37.3°C ± 1.3°C	0.595	37.4°C ± 1.2°C	37.3°C ± 1.5°C	0.671
Post-operative day 3 (mean ± SD)	37.4°C ± 1.1°C	37.3°C ± 1.6°C	0.647	37.1°C ± 1.3°C	37.2°C ± 1.4°C	0.661
Postoperative hospital stay (days ± SD)	7.12 ± 6.39	7.51 ± 7.17	0.698	7.02 ± 6.86	7.71 ± 7.79	0.581
30-day reoperation, *n* (%)	0	2 (0.68)	>0.999	0	0	-
30-day readmission, *n* (%)	2 (3.4)	11 (3.8)	>0.999	1 (1.9)	4 (3.7)	0.666
Medical cost (*yuan *± SD)						
Laparoscopic	70894.3 ± 2241	89912.6 ± 2873	0.132	71937.4 ± 2106	89644.6 ± 2923	0.276
Robotic	120773.7 ± 8796	131417.2 ± 5637	0.208	116030.2 ± 9022	120511.5 ± 4927	0.419

^a^ The highest body temperature.

### Histopathologic characteristic

Tumor size (2.9 ± 2.6 vs. 9.4 ± 4.2), proximal resection margin (2.5 ± 1.4 vs. 4.7 ± 3.8), distal resection margin (3.1 ± 1.9 vs. 12 ± 6.1) and number of dissected lymph nodes (18.31 ± 9.49 vs. 31.46 ± 15.61) were measured in the two groups, and the number of positive lymph nodes (2.1 ± 1.7 vs. 7.32 ± 3.17), pTNM stage and histological type were significantly different (*P* < 0.001). Notably, the TG group accounted for 82.2% of the histological type of poorly differentiated adenocarcinoma. There were no significant differences in tumor location or Lauren classification between the two groups (*P* > 0.05). After propensity matching, there was no significant difference in pTNM staging between the two groups (*P* > 0.05), and tumors in the PG group were significantly smaller than those in the TG group (2.3 ± 1.2 vs. 3.6 ± 2.1; *P* = 0.027). The proximal margin was smaller (2.1 ± 1.7 vs. 4.1 ± 3.6; *P* = 0.013), lymph nodes were removed (19.73 ± 10.03 vs. 24.75 ± 12.84; *P* = 0.023) and fewer were positive (1.9 ± 1.6 vs. 3.7 ± 4.1; *P* = 0.041), and all differences were statistically significant (all *P* < 0.05) (Table 3).

**Table 3:** Histopathologic characteristics.

Variable	Entire cohort		*P*	Matched cohort		*P*
	PG (*n* = 59)	TG (*n* = 292)		PG (*n* = 54)	TG (*n* = 108)	
Tumor location, *n* (%)			0.578			0.870
EG junction	15 (25.4)	57 (19.5)		14 (25.9)	22 (20.4)	
Cardia	5 (8.5)	27 (9.2)		5 (9.3)	9 (8.3)	
Fundus	21 (35.6)	94 (32.2)		18 (33.3)	41 (38.0)	
Upper body	18 (30.5)	114 (39.0)		17 (31.5)	36 (33.3)	
Tumour size (cm ± SD)	2.9 ± 2.6	9.4 ± 4.2	<0.001	2.3 ± 1.2	3.6 ± 2.1	0.027
Pathological proximal margin (cm ± SD)	2.5 ± 1.4	4.7 ± 3.8	<0.001	2.1 ± 1.7	4.1 ± 3.6	0.013
Pathological distal margin (cm ± SD)	3.1 ± 1.9	12 ± 6.1	<0.001	2.4 ± 1.5	11 ± 5.2	<0.001
Histological type, *n* (%)			<0.001			0.166
Poorly differentiated	36 (61.0)	240 (82.2)		32 (59.3)	77 (71.3)	
Moderately differentiated	18 (30.5)	43 (14.7)		18 (33.3)	28 (25.9)	
Well differentiated	4 (6.8)	2 (0.7)		3 (5.6)	1 (0.9)	
Undifferentiated	1 (1.7)	7 (2.4)		1 (1.9)	2 (1.9)	
Histology (Lauren classification), *n* (%)			0.907			0.892
Intestinal	17 (28.8)	82 (28.1)		15 (27.8)	29 (26.9)	
Diffuse	21 (35.6)	93 (31.8)		20 (37.0)	36 (33.3)	
Mixed	18 (30.5)	95 (32.5)		16 (29.6)	38 (35.2)	
Indeterminate	3 (5.1)	22 (7.5)		3 (5.6)	5 (4.6)	
T stage, *n* (%)			<0.001			0.989
T1a	23 (39.0)	62 (21.2)		22 (40.7)	42 (38.9)	
T1b	21 (35.6)	58 (19.9)		19 (35.2)	38 (35.2)	
T2	14 (23.7)	64 (21.9)		12 (22.2)	26 (24.1)	
T3	1 (1.7)	108 (37.0)		1 (1.9)	2 (1.9)	
N stage, *n* (%)			<0.001			0.215
N0	41 (69.5)	139 (47.6)		39 (72.2)	75 (69.4)	
N1	18 (30.5)	78 (26.7)		15 (27.8)	33 (30.6)	
N2	0	75 (25.7)		0	0	
pTNM stage, *n* (%)			<0.001			>0.999
IA	33 (55.9)	86 (29.5)		32 (59.3)	63 (58.3)	
IB	22 (37.3)	61 (20.9)		21 (38.9)	43 (39.8)	
IIA	4 (6.8)	41 (14.0)		1 (1.9)	2 (1.9)	
≥IIB	0	104 (35.6)		0	0	
Retrieved lymph nodes (mean ± SD)	18.31 ± 9.49	31.46 ± 15.61	<0.001	19.73 ± 10.03	24.75 ± 12.84	0.023
Positive lymph nodes (mean ± SD)	2.1 ± 1.7	7.32 ± 3.17	<0.001	1.9 ± 1.6	3.7 ± 4.1	0.041

### Operative complications and adverse events

Across the cohort, the severity of complications was classified by Clavien-Dindo and CTCAE version 5.0 classification. The number of early complications (i.e., complications occurring in the first 30 days after surgery) in both groups (PG group 12 vs. TG group 64; *P* = 0.788) and hematological adverse events (36 in PG group vs. 188 in TG group; *P* = 0.624) were evaluated. Postoperative reflux occurred in 6 patients in the PG group and 13 patients in the TG group (*P* = 0.076), and severe complications (grade iii or above) occurred in 3 patients in the PG group and 13 patients in the TG group. Among the hematological serious adverse events, there were 2 cases in the PG group and 11 cases in the TG group (grades 3–4). After matching, the number of complications in the two groups was 9 cases and 21 cases respectively (*P* = 0.668). Among the number of reflux cases, there were 6 cases in the PG group and 4 cases in the TG group (*P* = 0.133), showing no statistical significance. The number of adverse events was 32 cases and 71 cases (*P* = 0.419), among which, the number of anemia cases was 10 cases and 26 cases (*P* = 0.423), respectively, showing no statistically significant differences (Table 4).

**Table 4:** Postoperative morbidity and adverse events within 30 postoperative days.

Clavien-Dindo/CTCAE v5.0	Entire cohort		*P*	Matched cohort		*P*
	PG (*n* = 59)	TG (*n* = 292)		PG (*n* = 54)	TG (*n* = 108)	
Complications, *n* (%)			0.788			0.668
No	47 (79.7)	228 (78.1)		45 (83.3)	87 (80.6)	
Yes	12 (20.3)	64 (21.9)		9 (16.7)	21 (19.4)	
Clavien-Dindo Grade, *n* (%)			0.928			0.939
Grade I–II	9 (15.3)	51 (17.5)		8 (14.8)	17 (15.7)	
Grade III–IV	3 (5.1)	13 (4.5)		1 (1.9)	4 (3.7)	
Non-hematological, *n* (%)			0.948			0.856
Anastomotic leakage	0	2 (0.7)		0	0	
Anastomotic stenosis	0	2 (0.7)		0	1 (0.9)	
Cholecystitis	0	3 (1.0)		0	0	
Pancreatitis	1 (1.7)	4 (1.4)		0	1 (0.9)	
Pancreatic fistula	1 (1.7)	5 (1.7)		1 (1.9)	2 (1.9)	
Intraperitoneal hemorrhage	1 (1.7)	5 (1.7)		1 (1.9)	2 (1.9)	
Fluid abscess	0	7 (2.4)		0	1 (0.9)	
Wound infection	0	4 (1.4)		0	1 (0.9)	
Wound dehiscence	0	3 (1.0)		0	1 (0.9)	
Pneumonia	2 (3.4)	9 (3.1)		1 (1.9)	5 (4.6)	
Chyle leakage	1 (1.7)	5 (1.7)		0	2 (1.9)	
Regurgitation	6 (10.2)	13 (4.5)	0.077	6 (11.1)	4 (3.7)	0.133
Ileus	0	2 (0.7)		0	1 (0.9)	
Adverse events, *n* (%)			0.624			0.419
No	23 (39.0)	104 (35.6)		22 (40.7)	37 (34.3)	
Yes	36 (61.0)	188 (64.4)		32 (59.3)	71 (65.7)	
CTCAE v5.0 Grade, *n* (%)			0.898			0.658
Grade 1–2	34 (57.6)	177 (60.6)		31 (57.4)	67 (62.0)	
Grade 3–4	2 (3.4)	11 (3.8)		1 (1.9)	4 (3.7)	
Hematological, *n* (%)			0.997			0.950
Anemia^a^	13 (22.0)	74 (25.3)	0.591	10 (16.7)	26 (24.1)	0.423
Lymphocytopenia^b^	1 (1.9)	4 (1.4)		1 (1.9)	1 (0.9)	
Creatinine increased^c^	0	3 (1.0)		0	2 (1.9)	
Hypo-pre-albuminemia^d^	12 (20.3)	52 (17.8)		12 (22.2)	27 (25.0)	
Hyperbilirubinemia^e^	3 (5.1)	21 (7.2)		3 (5.6)	5 (4.6)	
AST/ALT increased^f^	3 (5.1)	14 (4.8)		2 (3.7)	2 (1.9)	
Hypernatremia^g^	0	1 (0.3)		0	0	
Hyponatremia^h^	3 (5.1)	14 (4.8)		3 (7.4)	7 (6.5)	
Hyperkalemia^i^	1 (1.9)	5 (1.7)		1 (1.9)	1 (0.9)	

^a^ Male patients Hb < 110 g/L, female patients Hb < 100 g/L.

^b^ Lymphocyte count < 1.1*10^9^/L.

^c^ Creatinine > 132umol/L.

^d^ Pre-albumin < 200 mg/L.

^e^ Total bilirubin > 22umol/L.

^f^ AST/ALT > 2.

^g^ Na > 147 mmol/L.

^h^ Na < 137 mmol/L.

^i^ K > 5.3 mmol/L.

### Clinical manifestations and nutritional status

There was no significant difference in clinical characteristics between the two groups 1 year after the operation. Overall, 26 patients in the PG group and 43 patients in the TG group (*P* = 0.312) reported no dietary problems. Reflux was present in 6 patients in the PG group and 3 patients in the TG group (*P* = 0.069), and there was no significant difference. In terms of nutrition score, although there was no statistical significance in different degrees of malnutrition between the two groups (*P* = 0.406), the PG group included a large proportion of mild malnutrition patients: 21 (38.9%) in the PG group and 33 (30.6%) in the TG group. Similarly, in the severe malnutrition patients, 2 (3.7%) were in the PG group. There were 9 patients in the TG group (8.3%) and a relatively small number in the PG group (Table 5).

**Table 5:** Comparison of postoperative clinical manifestations and nutritional score by PG-SGA between PG and TG 1 year after surgery.

	PG (*n *= 54)	TG (*n *= 108)	*P*
Symptom, *n* (%)			0.927
There are no dietary problems	26 (48.1)	43 (39.8)	0.312
Nausea	3 (5.6)	7 (6.5)	
Mouth pain	0	1 (0.9)	
A strange smell is scratching me	1 (1.9)	3 (2.8)	
Vomit	2 (3.7)	5 (4.6)	
Dry mouth	2 (3.7)	5 (4.6)	
No appetite	2 (3.7)	6 (5.6)	
Constipation	2 (7.4)	8 (7.4)	
Dysphagia	1 (1.9)	3 (2.8)	
Diarrhea	1 (1.9)	3 (2.8)	
Easy to fill	5 (9.3)	13 (12.0)	
It tastes tasteless or strange	1 (1.9)	3 (2.8)	
Abdominal pain	2 (3.7)	5 (4.6)	
Esophageal reflux	6 (11.1)	3 (1)	0.069
Overall evaluation, *n* (%)			0.406
Good nutritional status SGA-A (0–3)	21 (38.9)	33 (30.6)	0.289
Moderate or suspected malnutrition SGA-B (48)	31 (57.4)	66 (66.1)	0.650
Severe malnutrition SGA-C (>8)	2 (3.7)	9 (8.3)	0.440

**Figure 4:**
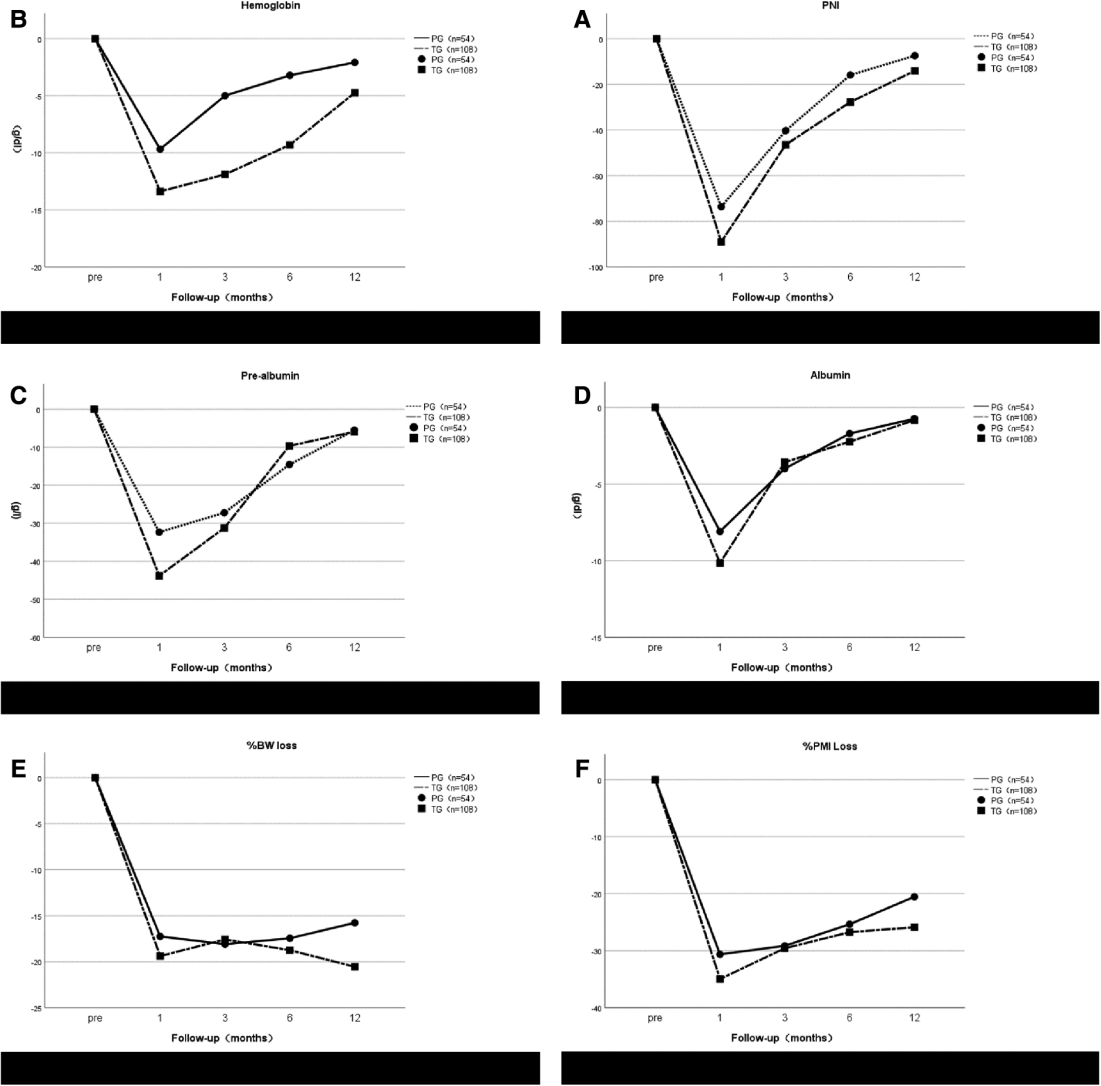
Postoperative changes of prognostic nutritional index (PNI) (**A**), hemoglobin (**B**), pre-albumin (**C**), albumin (**D**), %BW loss (**E**) and %PMI loss (**F**) in the proximal gastrectomy (PG) group and total gastrectomy (TG) group. All postoperative data are represented as values (mean ± standard error) relative to preoperative.

We assessed the rate of weight loss, the rate of Psoas muscle index loss, and changes in nutritional parameters in 162 patients followed for at least 1 year (Figure 4). The annual decrease in serum hemoglobin in the TG group was greater than that in the PG group, and the decrease in serum hemoglobin in the TG group at 3 and 6 months after surgery was significantly higher than that in the PG group (*P* = 0.032 and 0.046, respectively), There was no significant difference between the two groups at 1 and 12 months after surgery (*P* = 0.131 and *P* = 0.072, respectively). Regarding PNI, there was no significant difference between the PG group and the TG group (*P* > 0.05). However, the PNI decline in the TG group was always higher than that in the PG group after surgery. Although the TG group recovered faster from 1 to 3 months, the curves between the two groups have no intersection. Serum prealbumin levels in both groups were not significantly different at any time point (*P* > 0.05), but the TG group recovered faster at 3 to 6 months after surgery, and the levels in the two groups were almost the same at 12 months. Like albumin, there was no statistical significance at any time point, and the trend of change was not exactly the same as that of prealbumin. One month after surgery, %BW loss in TG group was significantly lower than that in the PG group (*P* = 0.024), and 6 months after surgery, %BW loss in the TG group was higher than that in the PG group. No significant differences were observed at any time point in %PMI loss between the two groups. No patients in either group died or relapsed during one year of follow-up.

### Reflux symptom and endoscopic findings

The patients will be graded according to the Visick score 1 year after operation, Visick score of reflux symptoms showed that there were 6 patients (11.1%) in the PG group and 3 patients (2.8%) in the TG group with grade ii or higher reflux symptoms (*P* = 0.069), which was not statistically significant. All patients underwent endoscopy approximately 1 year after surgery. These endoscopic findings were scored against the Los Angeles classification of reflux esophagitis and the results of preoperative endoscopy. In the PG group, 5 patients had grade A reflux esophagitis and 1 patient had grade B reflux esophagitis before surgery. After surgery, 7 patients developed grade A reflux esophagitis and 1 patient developed grade B reflux. In the TG group, grade A, B, and C reflux esophagitis occurred preoperatively in 1, 5, and 2 patients, respectively. After surgery, 4, 1, and 2 patients had grade A, B, and C reflux esophagitis, respectively (Tables 6, 7). After surgery, 8 patients (14.8%) in the PG group and 7 patients (6.5%) in the TG group had ≥A reflux (*P* = 0.085), showing no statistical significance.

**Table 6:** Reflux symptom scores 1 year after surgery in the propensity score-matched patients who underwent proximal or total gastrectomy.

	PG (*n* = 54)	TG (*n* = 108)	*P*
Visick score			0.079
I	48	105	0.069
II	5	2	0.076
III	1	1	>0.999
IV	0	0	>0.999

**Table 7:** Endoscopic findings at 1 year after surgery.

Reflux esophagitis	PG (*n* = 54)		TG (*n* = 108)	
LA grade	Preoperative	Postoperative	Preoperative	Postoperative
A	5	7	1	4
B	1	1	5	1
C	0	0	2	2
D	0	0	0	0

## Discussion

Proximal gastrectomy was introduced to improve patient performance status by conserving half of the stomach; thus, it is widely believed that proximal gastrectomy reduces postoperative weight loss. In addition, PG in the upper third of the stomach was believed to be appropriate in terms of both its radicality and safety (30, 31).

Our study showed that PG gastric tubular reconstruction had the advantage of less postoperative anemia and less %BW loss than TG. This result is consistent with previous reports (9, 32). Some studies have reported that PG with double-tract reconstruction does not have any advantages for postoperative anemia (28, 33, 34). Our data showed no significant difference in total protein and serum prealbumin. This result was consistent with previous reports (9, 35, 36). Based on its safety and simplicity, we believe that gastric tubular reconstruction can be a viable option after proximal PG. The use of a gastric tube provides a simple and safe anastomosis for PG because it is a single anastomosis. Kitano et al. introduced a reconstruction method using a gastric tube after PG (37). The authors indicated that the technique was simple and less invasive for EGC in the upper third of the stomach and evaluate the effectiveness of gastric tubular reconstruction to prevent reflux after open PG (38).

In 2017, Toyomasu reported that gastric tubular reconstruction has advantages, including being less invasive compared to jejunal interposition, shorter surgical duration, less surgical blood loss, and maintenance of postoperative nutritional status (39). Some previous reports showed different types of complications of PG. RE is common complication after PG. In the present study, RE with symptoms was diagnosed in 6 (11.1%) of 54 patients. This result was almost compatible with previous reports (40–43). But the rate of RE (≥Los Angeles grade A) has been reported to be over 30% (36, 44). Chen et al. (40) reported that only 14.3% of patients showed reflux symptoms after tube gastric anastomosis, and 57% of patients exhibited reflux esophagitis. Compared with traditional esophagogastric anastomosis, this method has obvious antireflux advantages. Aihara et al. (45) showed that 14% of patients had reflux symptoms after tube gastric anastomosis, while the incidence of anastomotic stenosis was 35%. Ronellenfitsch et al. (46) demonstrated that the incidence of reflux symptoms was 21.4% early (1–6 months) after esophagogastrostomy and 33.3% long (>6 months) after esophagogastrostomy. However, the symptoms were mild. Endoscopic examination results revealed that 29% of patients had esophagitis, and only 2 of them had reflux symptoms. Another study reported that after 3 weeks to 1 year follow-up, gastric tube anastomosis in patients with reflux symptoms was lighter than traditional residual stomach esophagus anastomosis; however, after 2–10 years of follow-up, there was no statistically significant difference the rate of reflux symptoms in patients with in gastric tube esophagus anastomosis compared with traditional residual stomach esophagus anastomosis (47).

The reflux symptoms of all patients in this study were graded 1 year after surgery using the Visick score. In total, nine patients had grade II reflux symptoms. Notably, the PG patient with Visick grade II reflux did not exhibit signs of reflux esophagitis on endoscopy 1 year after surgery: the Los Angeles scores were both grade 0. In contrast, the fifteen patients who exhibited reflux esophagitis on endoscopy 1 year after surgery (their grades ranged from A to C) all had Visick grade I scores. Thus, reflux symptoms did not correlate well with endoscopic findings. Several other studies have also reported this, both in patients who underwent gastrectomy and in patients with gastroesophageal reflux disease (46, 48). This may reflect differences between individuals in terms of sensitivity to subjective symptoms. Further prospective studies on the relationship between reflux symptoms and reflux esophagitis on endoscopy are needed.

Recently, several useful assessment scales and questionnaires have been developed to measure the subjective reflux symptoms of patients. They include Post gastrectomy Syndrome Assessment Scale (PGSAS-45), Functional Assessment of Cancer Therapy-Gastric (FACT-Ga), and European Organization for Research and Treatment of Cancer Quality of Life Questionnaire-Gastric Cancer (EORTC QLQ-STO22 and EORTC QLQ-C30). Future studies comparing surgical modalities for EGC should use these tools to assess the postoperative functional benefits of each modality (8, 49, 50).

Multiple studies have described the advantages of PG for treating this cancer. However, to date, few studies have assessed the usefulness of PG with gastric tube placement. In particular, the oncological safety of PG with gastric tube placement remains unclear due to the lack of long-term studies. For PG with gastric tube to become the standard surgical option for early proximal gastric cancer, it must be as oncologically safe as TG, offer a functional benefit, and be associated with minimal postoperative complications.

The oncological safety of proximal gastrectomy mainly involves the preservation of the supratropyloric, supratropyloric, distal lesser curvature of the stomach, and lymph nodes along the right perivascular gastroomentum. Studies have shown that there is no statistically significant difference in the overall postoperative survival rate between patients undergoing total gastrectomy and patients undergoing proximal gastrectomy for early upper gastric cancer (51). Therefore, the clinical oncology safety of proximal gastrectomy for early upper gastric cancer is not controversial, but for advanced upper gastric cancer, the oncology safety of proximal gastrectomy is still controversial. Oncology safety depends primarily on the impact of lymph node preservation on patient survival.

Yamashita et al. (52) showed that for esophageal and gastric junction carcinoma with tumor length <4 cm, the lymph node metastasis rates of Groups 4sa, 4sb, 4d, 5 and 6 were extremely low and were independent of tumor location and T stage. The results of a separate study of 202 patients with stage T2 and T3 proximal gastric cancer undergoing proximal gastrectomy demonstrated that the lymph node metastasis rates of Groups 4sa, 4sb, 4d, 5, 6, 8a and 12a were 3.47%, 1.49%, 0.99%, 0.00%, 0.00%, 2.02% and 0.006%, respectively, and the overall 5-year survival rate of the patients was 72.9%. Proximal gastrectomy was recommended for the treatment of T2 and T3 proximal gastric cancer (53). However, no prospective randomized controlled studies have been conducted on the long-term outcomes of total gastrectomy and proximal gastrectomy for locally advanced upper gastric cancer, and the oncological safety of proximal gastrectomy needs further clinical evidence.

There are several limitations to this study. First, the small number of patients in the cohort and the retrospective design of this study made the evidence of retrospective analysis less reliable than that of randomized controlled trials. These limitations were offset using propensity score matching to select a TG group that matched the PG group in terms of important baseline features. Even though propensity score matching was used, there is a selection bias according to the preference of the surgeon. However, surgery performed during the same period and the proficiency of the surgical method are not different. Second, the lack of long-term follow-up of nutritional status, assessment of patients’ long-term quality of life, and oncology outcomes largely limit the true benefits of PG. Third, we did not compare surgical methods in terms of postoperative VitB12 and serum iron, and anemia is a common complication of gastrectomy (54). Future studies comparing PG with tube gastric versus TG should examine postoperative iron panel blood test results. These studies should also determine the nutritional benefit of the surgical modalities by assessing the postoperative lipid profile. Finally, different reflux-scoring tools are needed to determine the effect of the surgeries on reflux symptoms.

## Conclusion

In this study, proximal gastrectomy with tubular gastrostomy was superior in clinical outcome to Roux-en-Y reconstruction for gastric cancer. These results suggest that PG combined with gastric tube anastomosis may be an appropriate surgical option for proximal gastric cancer. However, there is no standard procedure for early upper gastric cancer, and only prospective randomized trials in the future will clarify the true benefits of one procedure over another.

## Data Availability

The original contributions presented in the study are included in the article/Supplementary Material, further inquiries can be directed to the corresponding author/s.
